# Radiomics to Differentiate Malignant and Benign Breast Lesions: A Systematic Review and Diagnostic Test Accuracy Meta-Analysis

**DOI:** 10.7759/cureus.49015

**Published:** 2023-11-18

**Authors:** Ke En Oh, Nikhil Vasandani, Afiq Anwar

**Affiliations:** 1 Department of Surgery, University Hospital Galway, Galway, IRL; 2 Surgery, Royal College of Surgeons in Ireland, Dublin, IRL

**Keywords:** ai & robotics in healthcare, artificial intelligence in radiology, benign and malignant breast lesions, breast radiology, radiomics, ultrasound, mammogram, artificial intelligence, machine learning, deep learning

## Abstract

Breast cancer is a prevalent global health concern, necessitating accurate diagnostic tools for effective management. Diagnostic imaging plays a pivotal role in breast cancer diagnosis, staging, treatment planning, and outcome evaluation. Radiomics is an emerging field of study in medical imaging that contains a broad set of computational methods to extract quantitative features from radiographic images. This can be utilized to guide diagnosis, treatment response, and prognosis in clinical settings. A systematic review was performed in concordance with Preferred Reporting Items for Systematic Reviews and Meta-Analyses (PRISMA) guidelines and the Cochrane Handbook for Systematic Reviews of Diagnostic Test Accuracy. Quality was assessed using the radiomics quality score. Diagnostic sensitivity and specificity of radiomics analysis, with 95% confidence intervals (CIs), were included for meta-analysis. The area under the curve analysis was recorded. An extensive statistical analysis was performed following the Cochrane guidelines. Statistical significance was determined if p-values were less than 0.05. Statistical analyses were conducted using Review Manager (RevMan), Version 5.4.1. A total of 31 manuscripts involving 8,773 patients were included, with 17 contributing to the meta-analysis. The cohort comprised 56.2% malignant breast cancers and 43.8% benign breast lesions. MRI demonstrated a sensitivity of 0.91 (95% CI: 0.89-0.92) and a specificity of 0.84 (95% CI: 0.82-0.86) in differentiating between benign and malignant breast cancers. Mammography-based radiomic features predicted breast cancer subtype with a sensitivity of 0.79 (95% CI: 0.76-0.82) and a specificity of 0.81 (95% CI: 0.79-0.84). Ultrasound-based analysis yielded a sensitivity of 0.92 (95% CI: 0.90-0.94) and a specificity of 0.85 (95% CI: 0.83-0.88). Only one study reported the results of radiomic evaluation from CT, which had a sensitivity of 0.95 (95% CI: 0.88-0.99) and a specificity of 0.56 (95% CI: 0.45-0.67). Across different imaging modalities, radiomics exhibited robust diagnostic accuracy in differentiating benign and malignant breast lesions. The results underscore the potential of radiomic assessment as a minimally invasive alternative or adjunctive diagnostic tool for breast cancer. This is pioneering data that reports on a novel diagnostic approach that is understudied and underreported. However, due to study limitations, the complexity of this technology, and the need for future development, biopsy still remains the current gold standard method of determining breast cancer type.

## Introduction and background

Female breast cancer is the most commonly diagnosed cancer worldwide. It is one of the leading causes of cancer death among women, followed by colorectal and lung cancer [1]. In 2020, breast cancer surpassed lung cancer as the most commonly diagnosed malignancy, with an estimated 2.3 million new cases [1]. Over the past few decades, the management of breast cancer has progressed from radical treatment to targeted surgery with the use of more individualized therapeutic regimes [2]. In the era of precision medicine, early cancer diagnosis can allow for prompt medical intervention and effective treatment [3]. This will prevent the progression of disease from early to advanced stages. Consequently, this can help improve cancer prognosis and ultimately reduce overall mortality rates. Various imaging modalities are utilized in investigating breast cancer, including ultrasound sonography (USS), mammography (MMG), MRI, and CT. With the increasing incidence of breast cancer diagnosed, imaging is at the forefront of guiding clinicians in the best approaches to managing breast malignancies. In recent years, the rise of personalized medicine and the advancement of medical imaging analysis has encouraged the development of radiomics in the field of medicine. This exponential growth has enabled researchers to develop the field of radiomics through high-output computing. Radiomics is an emerging field of study in medical imaging that contains a broad set of computational methods. It uses data algorithms to extract quantitative features from radiographic images [4,5]. These extracted features, commonly known as radiomic features, can be utilized to guide diagnosis, treatment response, and prognosis in clinical settings. Radiomic analysis is built on the central hypothesis that tumor imaging reflects the underlying biological characteristics of the tumor, which may be presented as different radiomic values [5]. Previous studies have compared the performance of radiomics in several aspects of the diagnostic and staging process of many cancer pathologies, including breast cancer. This included differentiating benign and malignant breast lesions, predicting lymph node metastases from primary breast malignancies, identifying spinal metastases from lung primaries, predicting survival of patients with high-grade gliomas, and determining the invasiveness risk of stage 1 pulmonary adenocarcinomas. The exponential growth of radiomic research has led to its widespread integration into clinical practice. Radiomics in breast cancer strives to improve the understanding of the histopathology and treatment of breast tumors by extracting quantitative features from radiological images. The assessment of pre-operative imaging provides clinical information that may prove to be a practical alternative to diagnostic core tissue biopsy in stratifying breast cancers. Developing such methods of histopathological tumor evaluation would avoid invasive approaches until the time of surgery. This can help reduce patient anxiety and prevent potential complications associated with performing diagnostic core biopsies. In this study, we aim to perform a systematic review and meta-analysis of the diagnostic accuracy of radiomics in differentiating malignant and benign breast lesions using existing imaging modalities (MMG, USS, CT, and MRI). Our research question utilizes the population, intervention, comparison, and outcome (PICO) framework. Our population is patients diagnosed with breast cancer who have undergone imaging (MRI, CT, USS, and MMG). The proposed intervention involves a radiomic analysis of radiologic images of tumor tissue. The comparison is made between various radiomic software systems, Artificial Intelligence (AI), Machine Learning (ML), Conventional Neural Networks (CNN), Deep Learning Techniques (DLT), and the current gold-standard method of histopathological analysis via invasive biopsies. The primary outcome is to evaluate the clinical utility of imaging modalities in classifying breast cancer lesions as either benign or malignant. This evaluation will include an investigation of radiomic tumor characteristics, sensitivity, specificity, and area under the curve (AUC) scores from receiver operating characteristic (ROC) curve analyses. These strategies will help determine the reliability of radiomic imaging in confirming benign or malignant pathology, as validated by histopathological evaluation. The potential applications of radiomics in clinical research and practice are vast. It offers an alternative approach to diagnosing breast cancer, potentially reducing the need for traditional, more invasive methods. Such a shift could lead to a more comfortable and less painful diagnostic process, which is particularly beneficial for patients who may already be experiencing significant anxiety.

## Review

Materials and methods

A systematic review was conducted as per the Preferred Reporting Items for Systematic Reviews and Meta-Analyses (PRISMA) guidelines and in accordance with the Cochrane Handbook for Systematic Reviews of Diagnostic Test Accuracy [6,7]. Local institutional ethical approval was not required as this is a review article of the current literature. An electronic search of PubMed, Medline, EMBASE, and Scopus databases was performed for studies relevant to our research question. Only studies published in English were considered for inclusion and were not restricted based on the year of publication. All duplicate studies were removed before titles were screened. Studies deemed appropriate had their abstracts and full texts reviewed. This search was carried out by two independent reviewers. A third author was asked to arbitrate in case of a discrepancy between opinions. Studies deemed appropriate for inclusion were those applying radiomic AI to diagnostic imaging for differentiating benign from malignant breast lesions. These studies had to meet stringent inclusion and exclusion criteria for selection (Table 1). The rationale behind these criteria was to ensure that data were assessed from standard breast imaging modalities. Conventional breast imaging is highly informative in discriminating breast cancer pathological subtypes prior to surgery and in obtaining diagnostic biopsies. Additionally, ROC analysis plays an accepted role in providing diagnostic test accuracy, which is expressed in terms of sensitivity, specificity, and AUC.

**Table 1:** Inclusion and exclusion criteria for systematic review study selection. USS: Ultrasound sonography; MMG: Mammography; ROC: Receiver operating characteristic; AUC: Area under the curve.

Inclusion criteria	Exclusion criteria
Studies with patients who have histopathologically confirmed breast cancer and its associated subtypes	Studies reporting imaging modalities without application of radiomics
Studies including conventional methods of breast imaging (USS, MMG, CT, MRI)	Review articles and conference abstracts
Studies detailing ROC curve analysis (AUC, sensitivity and specificity)	Studies involving less than five patients in their patient cohort

Retrieved manuscripts were reviewed independently by both reviewers to ensure all inclusion criteria were met. Multiple data parameters were extracted from all selected studies, including first author name, year of publication, study design, country, level of evidence, study title, sample size, number of patients with breast lesions, benign lesions, malignant lesions, imaging modalities used, radiomic tumor properties evaluated, sensitivity, specificity, AUC scores from ROC analyses and type of radiomic analysis performed. Sensitivity, specificity, and 95% CI were directly extracted from tables and study text. The quality of each included study was assessed using the radiomics quality score (RQS), as outlined previously by Lambin P et al. [5]. An extensive statistical analysis was conducted following the Cochrane guidelines. Data on breast lesions were displayed using descriptive statistics. Estimations of sensitivity and specificity were calculated from the study data. ROC analysis was utilized to determine the sensitivity and specificity of radiomic analysis in confirming breast cancer subtypes as verified by pathology. Statistical significance was set at a P value of less than 0.05. All statistical analyses were performed using Review Manager (RevMan), version 5.4.1.

Results

**Figure 1:**
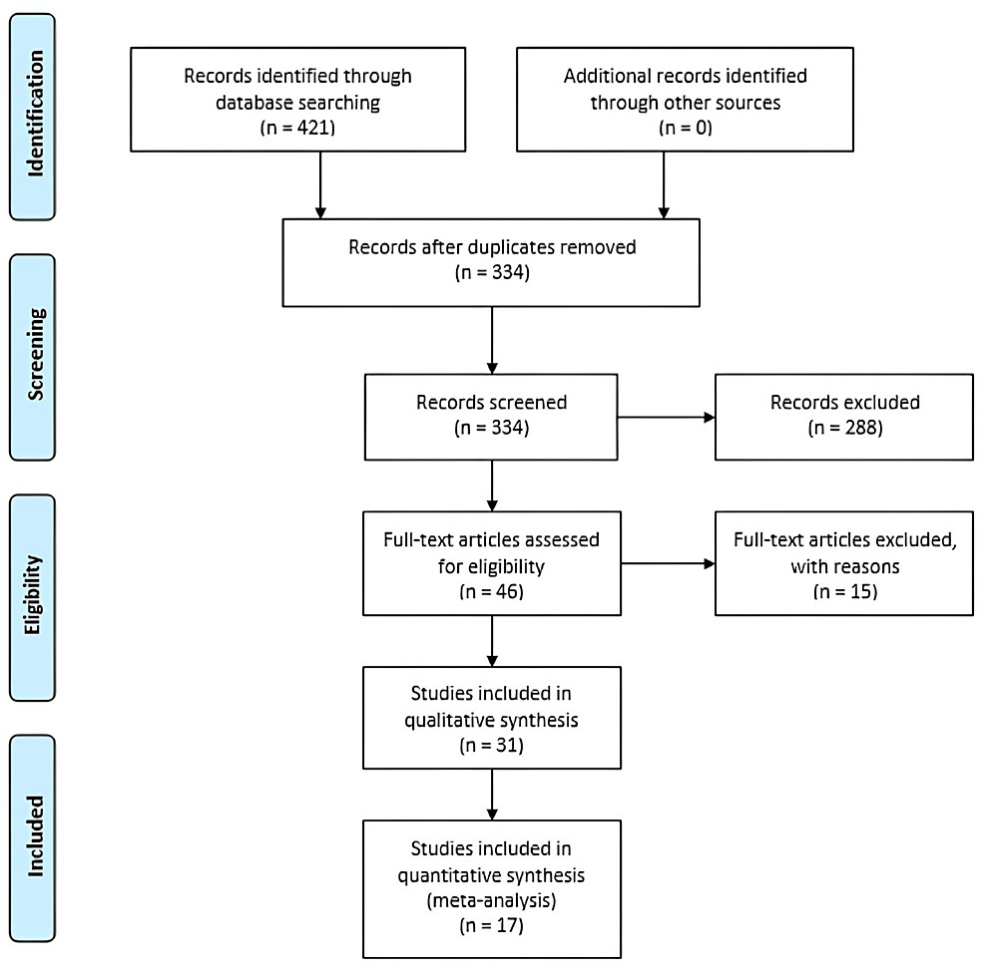
Preferred Reporting Items for Systematic Reviews and Meta-Analyses (PRISMA) flow diagram illustrating the number of studies screened and excluded.

An initial electronic search resulted in a total of 421 studies. Eighty-seven duplicate studies were found and removed. The remaining 334 studies were screened for relevance. Following the screening, 46 studies had their full text assessed for eligibility. Thirty-one manuscripts were included in this systematic review, and 17 were included in the meta-analysis (Figure 1) [8-38]. Studies included in this systematic review were taken from seven countries, of which 17 were from China (Table 2).

**Table 2:** Summary of 31 independent studies in this systematic review. PC: Prospective cohort; RC: Retrospective cohort; US: United States; SK: South Korea; N: Number of patients; NB: Number of breast lesions; MMG: Mammography; USS: Ultrasound sonography.

Study	Study	Country	Imaging Modality	N	NB	Benign	Malignant
Bickelhaupt S et al. (2016) [8]	PC	Germany	MRI	50	50	25	25
Bickelhaupt S et al. (2017) [9]	RC	Germany	MRI	51	51	22	29
Caballo M et al. (2020) [10]	RC	Netherland	CT	82	82	45	37
D'Amico NC et al. (2020) [11]	RC	Italy	MRI	68	68	41	27
Drukker K et al. (2019) [12]	PC	US	MMG	109	109	74	35
Ellmann S et al. (2020) [13]	RC	Germany	MRI	173	176	69	107
Fusco R et al. (2020) [14]	RC	Italy	MRI	59	59	26	33
Hu B et al. (2018) [15]	RC	China	MRI	88	88	36	52
Jiang Z and Yin J (2020) [16]	RC	China	MRI	192	192	93	99
Jin et al. (2020) [17]	RC	China	MRI	55	63	37	26
Kapetas P et al. (2019) [18]	PC	US	USS	124	124	59	65
Lee SE et al. (2018) [19]	RC	SK	USS	840	901	715	186
Li Z et al. (2017) [20]	RC	China	MMG	302	302	76	226
Li H et al. (2018) [21]	RC	China	MRI	112	112	67	45
Li Y et al. (2019) [22]	RC	China	USS	178	181	114	67
Lin et al. (2020) [23]	RC	China	MMG	139	139	100	39
Mao N et al. (2019) [24]	RC	China	MRI	76	80	40	40
Niu Q et al. (2018) [25]	RC	China	MRI	56	56	33	23
Parekh VS and Jacobs MA (2020) [26]	RC	US	MRI	138	138	41	97
Sakai A et al. (2019) [27]	RC	Japan	MMG	24	51	20	31
Wang L et al. (2020) [28]	RC	China	MMG	122	112	33	79
Wang L et al. (2020) [29]	RC	China	MMG	441	441	308	127
Wei M et al. (2020) [30]	RC	China	USS	448	448	184	264
Whitney HM et al. (2019) [31]	RC	US	MRI	508	508	212	296
Whitney HM et al. (2020) [32]	RC	US	MRI	3150	3150	745	2405
Yan C et al. (2022) [33]	RC	SK	USS	296	328	205	123
Yu Q et al. (2019) [34]	RC	China	MRI	251	274	154	120
Zhang Q et al. (2016) [35]	RC	China	USS	117	117	75	42
Zhang Q et al. (2020) [36]	RC	China	MRI	207	207	95	112
Zhou J et al. (2020) [37]	RC	China	MRI	133	153	62	91
Zhao Y et al. (2020) [38]	RC	China	MRI	184	207	119	88

A total of 8,773 patients were included in this study, with the mean age at diagnosis being 49.6 years ± 11.8 (ranging from 21 to 90 years). Breast lesions were reported in 8,967 cases. Of these, 5,036 cases were malignant breast cancers (56.2%), while 3,925 were benign (43.8%) (Table 2). Among the 21 studies that reported the histological subtype of breast cancer, the types included were invasive ductal carcinoma (IDC), invasive lobular carcinoma (ILC), ductal carcinoma in situ (DCIS), lobular carcinoma in situ (LCIS), fibroadenoma, and other benign and malignant histopathologies. IDC and fibroadenoma accounted for 29.2% and 36.7% of all cases, respectively (Table 3).

**Table 3:** Histopathology of breast lesions in 31 individual studies. IDC: Invasive ductal carcinoma; ILC: Invasive lobular carcinoma; DCIS: Ductal carcinoma in situ; LCIS: Lobular carcinoma in situ; MMG: Mammography; USS: Ultrasound sonography.

Study	Imaging Modality	IDC	ILC	DCIS	LCIS	Other invasive histology	Fibroadenoma	Other benign histology
Bickelhaupt S et al. (2016) [8]	MRI	-	-	-	-	-	-	-
Bickelhaupt S et al. (2017) [9]	MRI	26	2	1	0	0	6	16
Caballo M et al. (2020) [10]	CT	19	2	6	0	10	10	35
D'Amico NC et al. (2020) [11]	MRI	13	0	10	0	4	0	41
Drukker K et al. (2019) [12]	MMG	29	4	0	0	2	0	0
Ellmann S et al. (2020) [13]	MRI	0	0	47	13	47	19	50
Fusco R et al. (2020) [14]	MRI	17	6	10	0	0	15	11
Hu B et al. (2018) [15]	MRI	0	0	7	0	45	4	32
Jiang Z and Yin J (2020) [16]	MRI	93	1	5	0	0	35	58
Jin YN et al. (2020) [17]	MRI	20	0	5	0	1	23	14
Kapetas P et al. (2019) [18]	USS	53	6	4	0	2	24	35
Lee SE et al. (2018) [19]	USS	0	0	0	0	0	715	0
Li Z et al. (2017) [20]	MMG	154	30	30	0	12	51	25
Li H et al. (2018) [21]	MRI	32	2	7	0	4	6	61
Li Y et al. (2019) [22]	USS	58	0	2	0	7	50	64
Lin F et al. (2020) [23]	MMG	-	-	-	-	-	-	-
Mao N et al. (2019) [24]	MRI	27	2	10	0	1	12	28
Niu Q et al. (2018) [25]	MRI	-	-	-	-	-	-	-
Parekh VS and Jacobs MA (2020) [26]	MRI	29	12	34	22	0	10	31
Sakai A et al. (2019) [27]	MMG	-	-	-	-	-	-	-
Wang L et al. (2020) [28]	MMG	54	0	1	31	0	29	4
Wang L et al. (2020) [29]	MMG	-	-	-	-	-	-	-
Wei M et al. (2020) [30]	USS	-	-	-	-	-	-	-
Whitney HM et al. (2019) [31]	MRI	-	-	-	-	-	-	-
Whitney HM et al. (2020) [32]	MRI	-	-	-	-	-	-	-
Yan C et al. (2022) [33]	USS	-	-	-	-	-	-	-
Yu Q et al. (2019) [34]	MRI	96	16	0	0	8	98	56
Zhang Q et al. (2016) [35]	USS	-	-	-	-	-	-	-
Zhang Q et al. (2020) [36]	MRI	86	2	19	0	5	48	47
Zhou J et al. (2020) [37]	MRI	75	0	11	0	5	15	47
Zhao Y et al. (2020) [38]	MRI	119	0	0	0	0	88	0

In this analysis, 19 studies, constituting 61.3% of the total, reported data on the radiomic classification of breast cancer pathology using ML. Nine studies, representing 29%, utilized AI, while two studies employed deep learning techniques, and one study used a conventional neural network (Table 4).

**Table 4:** Radiomics and relevant imaging data of 31 independent studies included in this systematic review. MMG: Mammogram; US: Ultrasound; GE: General Electric; T: Tesla; N/R: Not reported; RQS: Radiomic Quality Score.

Study	Imaging Modality	Brand	Radiomic used	Radiomic software used	RQS
Bickelhaupt S et al. (2016) [8]	MRI	1.5T Philip Ingenia	Machine Learning	FMRIB, MiTK	20
Bickelhaupt S et al. (2017) [9]	MRI	1.5T MR Aera	Machine Learning	Matlab	17
Caballo M et al. (2020) [10]	CT	N/R	Machine Learning	N/R	19
D'Amico NC et al. (2020) [11]	MRI	1.5T Philip	Machine Learning	ITK	18
Drukker K et al. (2019) [12]	MMG	Senographe 2000D system	Machine Learning	N/R	22
Ellmann S et al. (2020) [13]	MRI	1.5T/3.0T Magnetom Avanto/Aera, Verio/Skyra	Machine Learning	RStudio 3.4.1	17
Fusco R et al. (2020) [14]	MRI	1.5 T Magnetom Symphony	Machine Learning	Matlab R2007a	17
Hu B et al. (2018) [15]	MRI	3T MagnetomVerio	Artificial Intelligence	A.K. software	15
Jiang Z and Yin J (2020) [16]	MRI	3T Signa HDxt	Machine Learning	MATLAB	18
Jin YN et al. (2020) [17]	MRI	3T GE Discovery 750	Artificial Intelligence	MADC, MaZda	16
Kapetas P et al. (2019) [18]	USS	Siemens Acuson S3000 US	Artificial Intelligence	VueBox	22
Lee SE et al. (2018) [19]	USS	iU22, HDI 5000	Artificial Intelligence	Matlab	17
Li Z et al. (2017) [20]	MMG	Hologic Selenia, GE Senographe Essential, Siemens Mammomat Inspiration	Machine Learning	Omni-Kinetics Version 2.1	16
Li H et al. (2018) [21]	MRI	N/R	Artificial Intelligence	N/R	18
Li Y et al. (2019) [22]	USS	Resona 7 US system	Artificial Intelligence	MATLAB R2016a	18
Lin F et al. (2020) [23]	MMG	Senographe DS Senobright	Artificial Intelligence	N/R	20
Mao N et al. (2019) [24]	MRI	3T MR750	Machine Learning	Omini-Kinetic	17
Niu Q et al. (2018) [25]	MRI	3T MR750	Machine Learning	Omni-Kinetic	17
Parekh VS and Jacobs MA (2020) [26]	MRI	3T Philip	Machine Learning	Matlab	16
Sakai A et al. (2019) [27]	MMG	AMULET Innovality	Machine Learning	N/R	16
Wang L et al. (2020) [28]	MMG	N/R	Artificial Intelligence	AK	21
Wang L et al. (2020) [29]	MMG	Selenia Dimensions mammography	Neural Networks	DenseNet	18
Wei M et al. (2020) [30]	USS	PHILIPS iu22, PHILIPS iu Elite	Machine Learning	N/R	18
Whitney HM et al. (2019) [31]	MRI	3T Philip	Machine Learning	ROCkit	19
Whitney HM et al. (2020) [32]	MRI	Philip, GE Discovery 750	Machine Learning	ROCkit	18
Yan C et al. (2022) [33]	USS	SuperSonic Imagine	Machine Learning	MATLAB R2010a	18
Yu Q et al. (2019) [34]	MRI	3T Philips Achieva	Machine Learning	N/R	17
Zhang Q et al. (2016) [35]	USS	Hitachi HI VISION Preirus	Machine Learning	N/R	17
Zhang Q et al. (2020) [36]	MRI	3T Ingenia	Artificial Intelligence	Omni-Kinetic	18
Zhou J et al. (2020) [37]	MRI	3T GE SIGNA HDx	Deep Learning	ResNet50	22
Zhao Y et al. (2020) [38]	MRI	1.5 T Achieva	Deep Learning	KERAS	19

**Figure 2:**
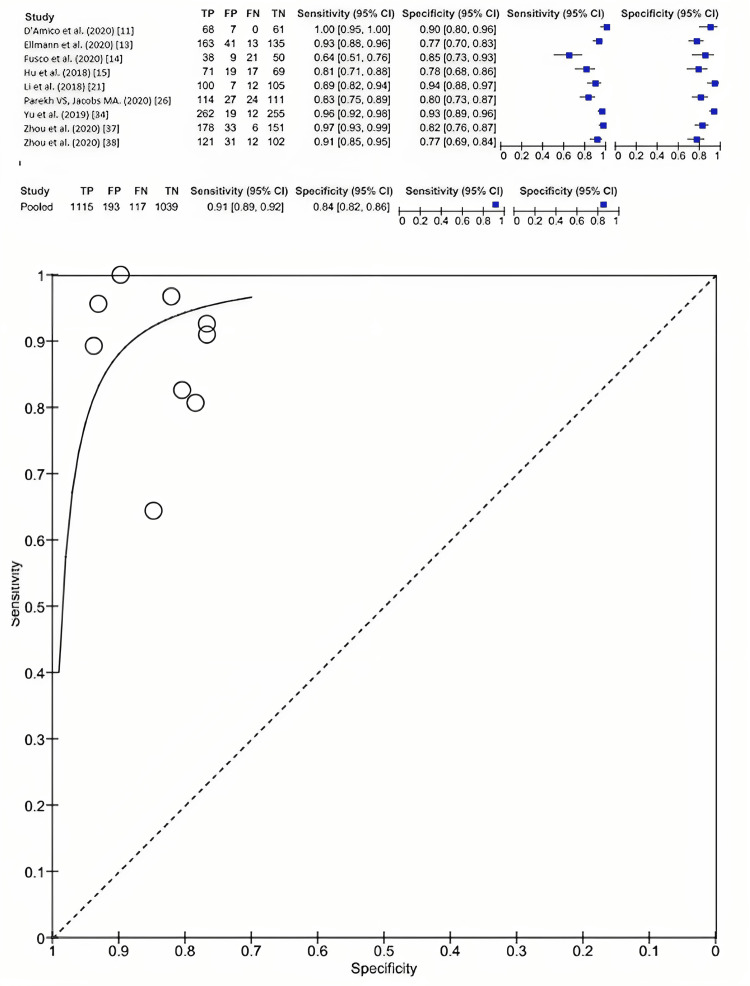
The diagnostic test accuracy of radiomic features in MRI imaging for differentiating between benign and malignant breast cancer.

**Figure 3:**
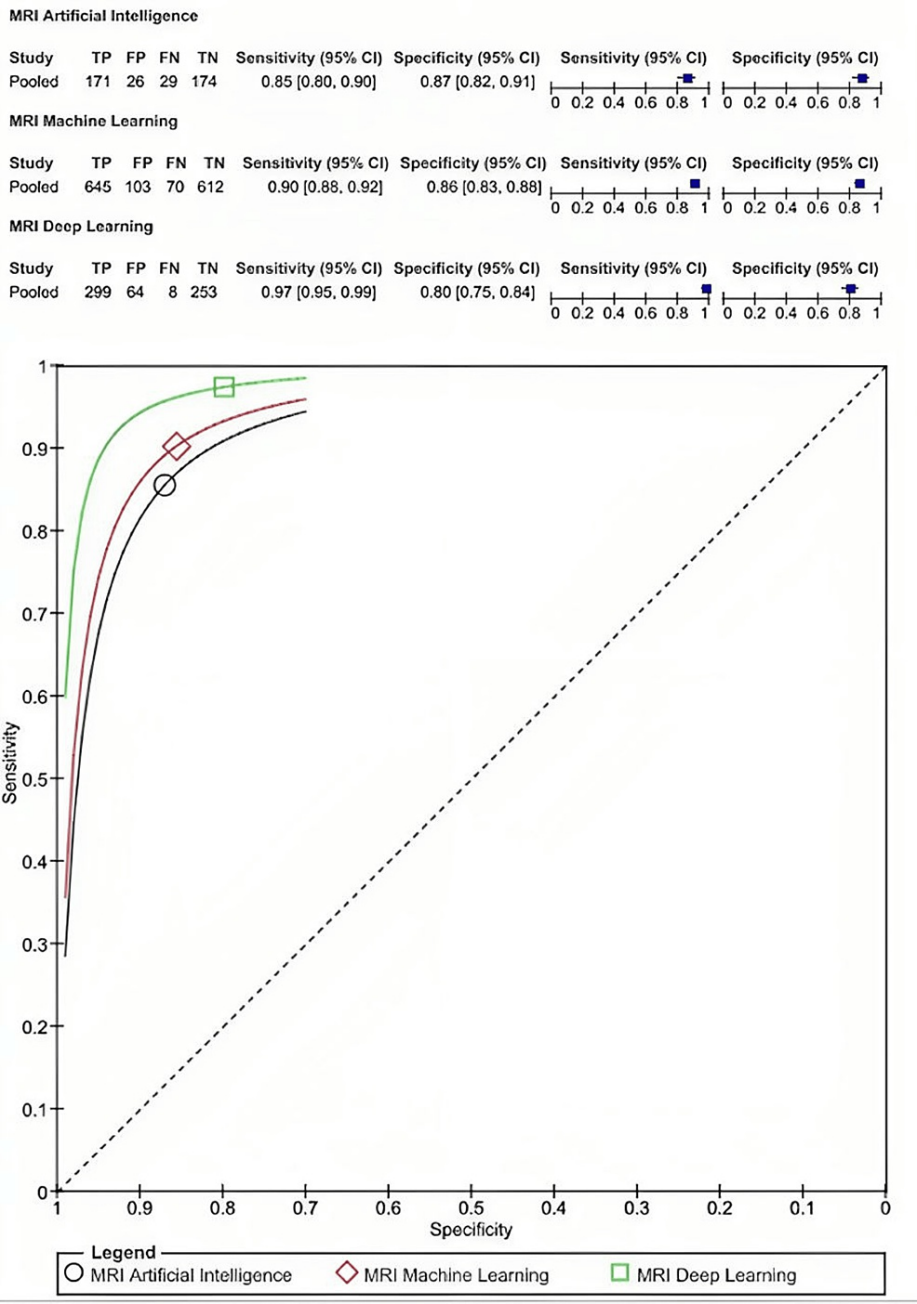
The comparison of diagnostic test accuracy of radiomic features in MRI imaging among Artificial Intelligence, Machine Learning, and Deep Learning techniques.

Overall, nine included studies reported the value of radiomic properties from MRI in estimating breast cancer pathology subtype. Evaluation of radiomic features on MRI in differentiating malignant and benign cancer had a sensitivity of 0.91 (95% CI: 0.89-0.92] and specificity of 0.84 (95% CI: 0.82-0.86) (Figure 2). Deep learning techniques augment and improve the sensitivity to 0.97 (95% CI: 0.95-0.99) compared to both AI and ML, which had a sensitivity of 0.85 (95% CI: 0.80-0.90) and 0.90 (95% CI: 0.88-0.92), respectively. However, deep learning techniques exhibited a lower specificity of 0.80 (95% CI: 0.75-0.84) compared to AI, which had a specificity of 0.87 (95% CI: 0.82-0.91), and ML, with a specificity of 0.86 (95% CI: 0.83-0.88) (Figure 3).

**Figure 4:**
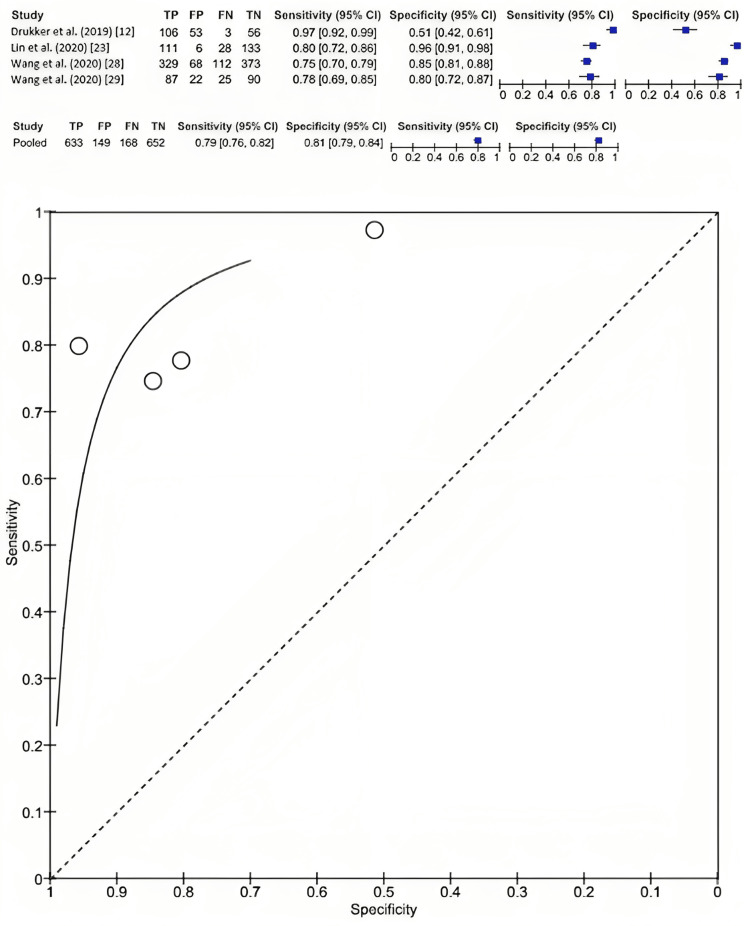
The diagnostic test accuracy of radiomic features in mammography for differentiating between malignant and benign breast cancer.

Overall, four included studies reported on the value of radiomic features from mammographic imaging in predicting the subtype of breast cancer. The evaluation of these radiomic features in mammography for differentiating between malignant and benign cancers demonstrated a sensitivity of 0.79 (95% CI: 0.76-0.82) and a specificity of 0.81 (95% CI: 0.79-0.84) (Figure 4).

**Figure 5:**
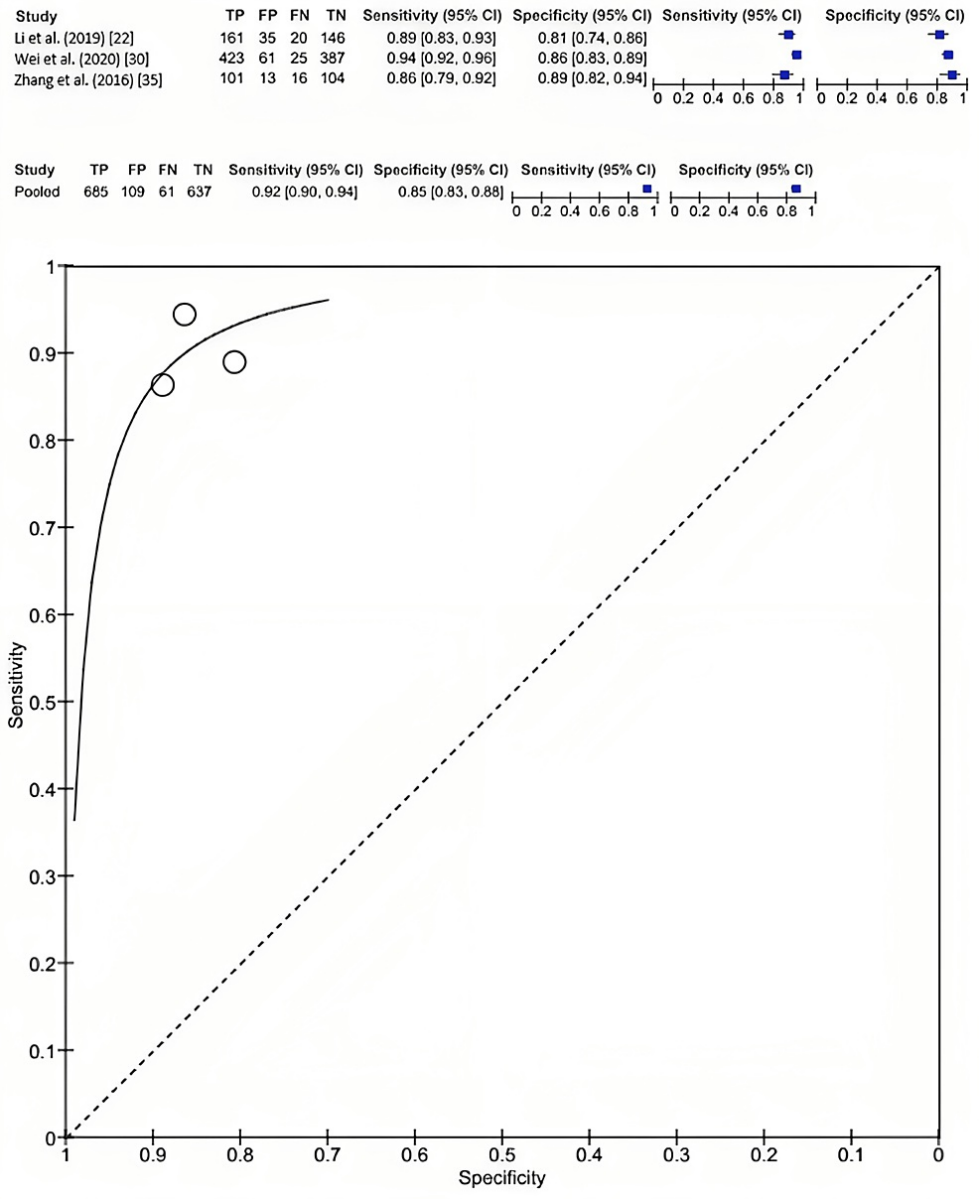
The diagnostic test accuracy of radiomic features in ultrasound sonography for distinguishing between malignant and benign breast cancer.

Overall, three included studies reported the value of radiomic features derived from ultrasound imaging in predicting the pathology of breast cancer. The appraisal of these radiomic features on ultrasound for differentiating malignant from benign breast cancer demonstrated a sensitivity of 0.92 (95% CI: 0.90-0.94) and a specificity of 0.85 (95% CI: 0.83-0.88) (Figure 5).

Only one study, by Caballo M et al., reported the results of radiomic evaluation using CT, which demonstrated a sensitivity of 0.95 (95% CI: 0.88-0.99) and a specificity of 0.56 (95% CI: 0.45-0.67).

Discussion

Current systematic reviews and meta-analyses in the literature present comparable diagnostic test accuracy in differentiating benign and malignant breast lesions using radiomic analysis compared to diagnostic core biopsy. This study portrays a strong detectability of radiogenomic analysis in the stratification of breast cancer into benign and malignant groups. Preoperative radiomic data from over 8,000 female patients diagnosed with breast cancer was utilized in the study. This is the first of its kind in the radio-oncologic literature. A myriad of studies focused on the use of radiomics in tumor analysis were identified. Most of these studies were published in the last three years, making them relevant in modern literature. Data from this analysis demonstrates the potential of radiogenomic analysis of diagnostic imaging in identifying benign and malignant breast lesions, with an AUC of over 80%. Generally, the work-up of newly presenting breast lesions involves the standard 'triple assessment' approach. This includes clinical examination, imaging, and biopsy of any identified lesions [39-41]. This is the foundation of preoperative assessment and work-up of symptomatic breast patients. Diagnosis and ultimate management strategy of breast lesions rely heavily on their histopathology and genetic profile. This can be increasingly onerous. A multidisciplinary review of data retrieved from a standard triple assessment allows for more personalized treatment. The current gold standard core tissue biopsy utilizes an invasive approach to acquire preoperative diagnostic tissue [42]. This approach is limited due to the heterogeneous nature of tumors and due to continuous mutation [43, 44]. Radiomics employs a non-invasive and reproducible process of extracting quantitative tumor features from radiological imaging. This allows for a more streamlined single assessment and evaluation approach for each tumor [45]. As a result, radiomics can be viewed as a novel method of diagnosis for breast cancer patients. According to this study, it can be extrapolated that the application of radiomics in MRI is a reliable predictor in determining the histopathological type of a breast lesion. MRI has a high sensitivity in diagnosing invasive breast cancer [46-49]. Breast MRI is widely used for high-risk screening, monitoring patients on neoadjuvant chemotherapy, and for local staging. However, its widespread use can lead to false positive results and, ultimately, overdiagnosis [50,51]. Radiomics can circumvent these limitations. Regarding radiomic methods, deep learning techniques boost and enhance the sensitivity compared to AI and ML [8-27]. In regards to the type of breast lesion, our analysis substantiates the use of deep learning and CNN. These have been thought of as sophisticated networks as opposed to more basic AI methods. CNN serves as an automated method of backpropagation utilizing multiple building blocks to adaptively learn spatial hierarchies of features [52]. These building blocks include convolution, pooling, and fully connected data processing layers [52]. Due to the complexity of layered data points presented by MRI, CNN would prove to be beneficial. Conversely, deep neural networks are vulnerable to adversarial examples. The implication of this susceptibility is crucial, yet unknown, in medical imaging [53]. Adversarial examples are carefully chosen inputs that cause the network to change output without a visible change to a human, with artificial networks having a different means of seeing and predicting [53]. Its implication in medical imaging is relevant since the clinical application of deep learning requires robustness before it can be used in patients as opposed to relatively trivial non-medical tasks. MMG is the conventional diagnostic imaging modality in breast cancer screening programs worldwide. It has sensitivity and specificity rates of over 85% [54]. Early detection of breast cancer reduces mortality rates, improves prognosis, and reduces overall disease burden. In this analysis, the performance of radiomics in MMG offers a positive result. The addition of radiomic analysis to diagnostic MMG can be seen as a more reliable screening tool with the potential to expedite breast cancer stratification.

Furthermore, compared to MRI, this analysis has intriguingly identified radiomic assessment in USS as a strong predictor in characterizing benign and malignant breast lesions. Breast density and age are important factors in determining the accuracy of USS [55]. Notably, USS has been found to be more accurate than MMG in symptomatic women under the age of 45 [55]. The integration of radiomics into diagnostic USS could further enhance the speed of diagnosis and treatment decision-making. This advancement holds the potential to accelerate the development of personalized management plans for this particular demographic.

Radiomic analysis could play a significant role in detecting invasive micropapillary breast cancers (IMPC). This is a rare subtype that is often discussed for its potency for lymphovascular invasion and difficulty in accurate imaging estimation. The mammographic appearance of IMPC is frequently nonspecific, with 66.7% often misclassified as IDCs or DCIS. Mammographic assessment tends to consistently underestimate disease size, with reported false-negative rates as high as 12% for IMPC patients. Additionally, USS often fails to capture the true depth of IMPC tumor invasion, resulting in a substantial false-negative rate of up to 47%. However, when identified, the actual extent is reported to be underestimated in 81% of cases. While MRIs prove most effective in distinguishing IMPC, there is still a notable probability of finding non-mass enhancement lesions. Despite MRI's superiority, there remains a likelihood of missing lesions, particularly diffuse multifocal lesions with extensive DCIS. In light of these challenges, radiomic analysis emerges as pivotal in distinguishing IMPCs. ML and AI models can be trained to help better identify IMPCs from USS, mammograms, and MRIs, ultimately offering valuable support for more accurate diagnoses [56]. It was hypothesized that radiomic breast cancer analysis is a minimally invasive approach that accurately categorizes breast lesions into operative and non-operative cohorts. The following study supports the following statement by proving that radiomics is a novel method that has the potential to rapidly and efficiently label differing breast lesions into benign and malignant groups. In the interim, we acknowledge that this technology is still new, requires refinement, and that this review comes with a significant number of limitations. We conclude that this should not supersede the current gold standard of performing diagnostic core biopsies despite early promising results of performing radiomic analyses.

One of the study's main limitations is that the correlation of extracted individual radiomic features from the studies into pathology was insubstantial. Radiomic features are not included nor addressed, even though there was the potential for crucial differences to be introduced into the results if they had been addressed. This resulted in the reliance on meta-analysis techniques solely to provide insight into radiomics and its role in differentiating benign from malignant breast lesions. Radiomics encompasses a spectrum of AI methods. This includes ML, CNN, and deep learning techniques, with variance in data reproducibility based on the method employed in individual papers. However, this analysis utilized the umbrella term 'radiomics' to appraise all these methods despite the abovementioned variance. Moreover, the study encompasses various imaging modalities, including MRI, mammography, ultrasound, and CT. A wide variation in sample sizes amongst included studies was also noted. The variation in imaging techniques and sample sizes might introduce heterogeneity in the data, which can ultimately impact the validity of the results. Additionally, the majority of the studies included were from China. The limited geographical diversity amongst selected papers could limit the generalizability of the findings to a more diverse population. In such cases, we could have adopted bootstrapping or cross-validate parts of the data to circumvent this limitation. Furthermore, the selected studies present a wide variety of sample sizes, with most studies relying on small sample sizes. Larger multicenter data is needed in further studies to improve our evaluation. In addition, this study is further limited by the fact that only a few studies had prospective designs.

Despite breast USS being the standard imaging protocol in evaluating patients with suspicious breast masses, only three studies looked at using USS radiomics to delineate benign and malignant breast lesions. USS, compared to modalities such as MRI, has an essential role in routine patient work-up and could potentially serve as a more clinically pragmatic and relevant source of radiomic quantitative and qualitative data. However, the use of USS is operator-dependent, whereas MRI often provides false positive findings. This can ultimately lead to performing unnecessary biopsies.

In current practice, implementing radiomic software and hardware remains an arduous process due to its innate complexity. In order to overcome these challenges practically, investment from large multinational companies and government entities is necessary.

## Conclusions

Data from this systematic review and meta-analysis support the use of radiomic analyses of preoperative diagnostic imaging in differentiating benign from malignant breast lesions. However, this approach is limited by the fact that the sensitivity and specificity rates of radiomics rarely surpass 95%. This indicates that improvements in existing radiomic techniques are required before they can be implemented as a robust adjunct to current diagnostic modalities.

Additionally, this analysis supports using deep learning and CNN methods as the most favorable techniques for performing a radiomic analysis. Tissue biopsies are the current gold standard in breast cancer diagnostics. These are limited through its lack of representativeness of the tumor as an entirety. This restricts the process of ascertaining a thorough understanding of tumor behavior and, subsequently, treatment response.

As the paradigm shifts towards precision medicine and personalized cancer therapeutics, future radio-oncological research may develop radiomic techniques capable of predicting disease. As a result, there is scope for more effective and less toxic therapies to be developed and employed, providing a tremendous benefit to cancer patients.

In conclusion, radiomics has the potential to offer a significant contribution towards the diagnostic work-up of breast lesions. This review contributes interesting and pioneering data to current literature and clinical practice. It highlights a novel, minimally invasive adjunct to current breast cancer diagnostics that is understudied and underreported and has the potential to change future practice.
